# Upregulation of *SPP1* Is a Marker for Poor Lung Cancer Prognosis and Contributes to Cancer Progression and Cisplatin Resistance

**DOI:** 10.3389/fcell.2021.646390

**Published:** 2021-04-29

**Authors:** Huaping Tang, Jianyou Chen, Xiaolei Han, Yan Feng, Fang Wang

**Affiliations:** ^1^Department of Pulmonary and Critical Care Medicine, Qingdao Municipal Hospital, Qingdao, China; ^2^Health Office, Qingdao Municipal Hospital, Qingdao, China

**Keywords:** *SPP1*, lung cancer, *DNMT1*, cisplatin resistance, chemotherapeutic drug

## Abstract

The chemoresistance of lung cancer is a significant contributor to its high mortality and morbidity rate. There is an urgent need to identify differentially expressed genes in lung cancer patients with a poor prognosis to develop effective means to overcome drug resistance in subsequent treatment. In this study, we identified the secreted phosphoprotein 1 (*SPP1*) as a potential gene associated with a poor diagnosis of lung cancer patients using the Cancer Genome Atlas analysis, which suggested that the expression of *SPP1* in tumor tissues was significantly higher than normal tissues. The high expression of *SPP1* was also correlated with tumor grade and poor clinical prognosis. To understand the roles of *SPP1* and the DNA methyltransferase 1 (*DNMT1*), which regulated *SPP1* expression, in affecting cell viability, migration and invasion, *SPP1* and *DNMT1* were overexpressed in the human lung cancer A549 and NCI-446 cells, followed by analyzing cell viability, migration and invasion. We showed that *SPP1* promoted the proliferation, migration and invasion of lung cancer cells, and increased the resistance of lung cancer to the chemotherapeutic drug cisplatin. Knocking down *SPP1* in cells restored sensitivity to cisplatin. Further, A549 cells without *SPP1* overexpression demonstrated lower tumor growth rate than *SPP1* overexpression cells using the xenograft tumor mouse model. High expression of *SPP1* in lung cancer tumor tissue was caused by the reduced methylation level of its promoter region mediated by *DNMT1*. Our data suggested that *SPP1* can be used as a marker for highly malignant lung cancer and targeting *SPP1* may be a potential lung cancer treatment strategy.

## Introduction

Lung cancer is the first leading cause of cancer-related death, and a large portion of patients are diagnosed at an advanced stage (Torre et al., 2016). Lung cancer is particularly prone to develop drug resistance during treatment, which further aggravates the survival of these patients (Qin et al., 2017). Cisplatin is one of the first-line drugs for lung cancer. However, lung cancer with cisplatin resistance is common and there is a lack of treatments against lung cancer because of drug resistance (Qin et al., 2017). Therefore, it is urgent to find differentially expressed genes in lung cancer patients as a marker for early screening in clinical practice (Wang and Chen, 2020), and to find effective means to overcome drug resistance in subsequent treatment.

Over recent years, a number of genes have been identified to be linked to the resistance of lung cancers, which leads to the development of novel biomarkers for lung cancer diagnosis and treatment (Vargas and Harris, 2016). The Cancer Genome Atlas (TCGA) analysis is a novel tool to facilitate the identification of differentially expressed genes associated with lung cancer malignancy (Silva et al., 2019). The use of TCGA analysis, incorporated with *in vitro* and *in vivo* validation of the identified markers, has become a powerful tool in driving technology advancement of cancer treatment (Wang and Chen, 2020).

Herein, we utilized TCGA analysis to screen for biomarkers of aggressive and drug-resistant lung cancers. *SPP1* gene (encoding secreted phosphoprotein 1) was identified as a pronouncedly upregulated gene in drug-resistant lung cancer (Zhang et al., 2017). Secreted phosphoprotein 1 is an extracellular matrix (ECM) protein, which interacts with a number of adhesion receptor binding motifs including a C-terminal CD44v6 domain and thrombin-cleaved N-terminal integrin domains (Zeng et al., 2018). *SPP1* gene is therefore involved in cell adhesion and other similar signaling pathways and promotes tumor progression and metastasis. *SPP1* gene is reported to participate in many pathological and biological processes, including inflammation, tissue remodeling, immunity angiogenesis, tumor development and metastasis. Previously, the upregulation of *SPP1* was found to be linked to gastric cancer (Junnila et al., 2010), osteosarcoma (Dalla-Torre et al., 2006), ovarian cancer (Zeng et al., 2018), oral squamous cell carcinoma (Huang et al., 2014), etc. *SPP1* upregulation was also found to be linked to immune escape of lung cancer (Zhang et al., 2017). However, the role of *SPP1* in lung cancer is yet to be clarified.

In this study, we set force to verify the specific role of *SPP1* in regulating lung cancer resistance and lung cancer cell progression. Further, as a mechanistic study, we hypothesized that DNA methylation was responsible for regulating the expression of *SPP1* in lung cancer. The results of the study could provide insight on *SPP1* as a potential new diagnostic and therapeutic target in lung cancer.

## Materials and Methods

### TCGA Analysis

TCGA analysis was performed according to standard procedures in the dataset provided by http://ualcan.path.uab.edu/. Data of gene expression were acquired using the Illumina HiSeq platform, followed by quantification using RSEM.

### Cell Culture and Transfection

We used the A549 and NCI-446 cells in the study to elucidate the role of *SPP1* in human non-small cell and small cell lung cancer cell lines, respectively. These two commonly used cell lines, which differ in cell sizes, are derived from human adenocarcinoma and have a high metastatic capability. The A549 and NCI-446 cells, acquired from American Type Culture Collection (Rockville, MD, United States), were cultured in DMEM medium supplemented with 10% fetal bovine serum. The cells were incubated at 37°C and 5% CO_2_. The A549 and NCI-446 cells were treated with 10 μM cisplatin for 6 months to generate the cisplatin resistant cell lines.

Expression plasmids for human *SPP1* (isoform 5) and human DNA methyltransferase 1 (*DNMT1*) gene ectopic overexpression, pcDNA3.1-*SPP1* and pcDNA3.1-*DNMT1*, and siRNA specific to *SPP1*, si*SPP1*, were constructed by Integrated Biotech Solutions (Shanghai, China). The sequence of si*SPP1* was: UAUUUUGGCCUUUAUUCUGUU. Non-targeting siRNA (cat#4390843, ThermoFisher Scientific) severed as negative control (NC). Transfection of cells was performed using Lipofectamine 2000 (Invitrogen, United States).

### Viability Assay

Cell counting assay and MTT assay were used to measure the cell viability. Cell counting was performed using a cell counter. MTT assay was performed after plating 2 × 10^3^ cells in each well and after 24 h, cells were added with 10 μL of MTT agent (Sigma, St. Louis, MO, United States) and incubated for another 4 h. Following this, cell medium was removed and 20 μL DMSO was added to dissolve the precipitates and a plate reader was used to measure the absorbance at 480 nm.

### Transwell Assay

The transwell assay was used to evaluate cell migration and invasion using a transwell apparatus according to the manufacturer’s recommendations (Corning, United States). Cells (2 × 10^5^) were seeded in the upper chamber of the apparatus. For invasion assay, the bottom of the upper chamber was coated with Matrigel (1:8 diluted), while for migration assay the bottom was not coated. The upper chamber was added with serum-free medium (0.2% BSA) and the lower chamber was added with 20% FBS medium. After incubation for 24 h, the cells in the lower chamber were fixed and stained with trypan blue. The positively stained cells were manually counted. The cells in three random fields of one chamber were counted to calculate the average cell number.

### Quantitative Real-Time PCR

RNA from cells was isolated using the Trizol reagent (Invitrogen, Cambridge, MA, United States). RNA was reversely transcribed into cDNA using the cDNA synthesis kit (Abcam, United States), and real-time PCR was conducted with the SYBR green reagent (Invitrogen, United States). The primers used in this study included: *SPP1* (isoform 5): forward-AGTCCAGATTATATAAGCGGAA; reverse-CTTTTGGGGTC TACAACCAG; DMNT1: forward-GAACCAGAGATGTTAAC CGAT; reverse-GCACAACTAAAGTACAGTTCCAC. GAPDH: forward-AGGGCTGCTTTTAACTCTGGT; reverse-CCCCAC TTGATTTTGGAGGGA. GAPDH was used as an internal control, and the relative quantitative value was expressed as 2^–ΔΔCt^.

### Western Blot and ELISA

Protein lysates were collected from cells were loaded onto 12% SDS-PAGE for electrophoresis, followed by transferring to PVDF membranes. After blocking with 5% fat milk, the membranes were incubated with primary antibodies against *SPP1* or GAPDH (loading control) dissolved in TBS-Tween20 (TBST, 0.1% v/v) and incubated in room temperature for 1 h. After washing with TBST three times, HRP-conjugated secondary antibodies were incubated with the membrane overnight at 4°C. All antibodies were acquired from Abcam using recommended dilutions. The proteins were imaged after adding the enhanced chemiluminescence agent (Abcam) and images were captured. Protein expression was normalized to GAPDH. The soluble *SPP1* protein levels in the supernatant medium were determined by *SPP1* ELISA kit (RD system) as the manufacturers indicated.

### Tumor Growth Study

The female Balb/c nude mice (8–12 weeks, body weight 22 ± 2 g) were purchased from Jackson Laboratories (Bar Harbor, ME, United States). Each group of mice (*n* = 5) was housed in standard cleaned cages in a room with humidity of 60%, temperature of 22 ± 2°C, and lighting of 12:12-h light-dark cycle. A549 cells (1 million per mouse) with or without *SPP1* overexpression were subcutaneously injected into flanks of mice. A caliper was used to measure the width and length of the tumors, and tumor volume was calculated as width^2^ × length/2. Animal studies were conducted in strict accordance with the institutional guidelines, and approved by Qingdao Municipal Hospital (2019-a7).

### Statistical Analysis

Data were presented as mean ± standard deviation (SD) and analyzed by SPSS (Chicago, IL). Multi-group comparisons were performed using one or two-way ANOVA test followed by an appropriate *post hoc* test, and two-group comparisons were performed using Student’s *t*-test. Kaplan-Meier analysis was used for analyzing survival data. All the experiments were performed at least three times as independent biological replicates. *P* < 0.05 was considered statistically significant.

## Results

### Highly Malignant Lung Cancer Is Characteristic of *SPP1* Upregulation

**FIGURE 1:**
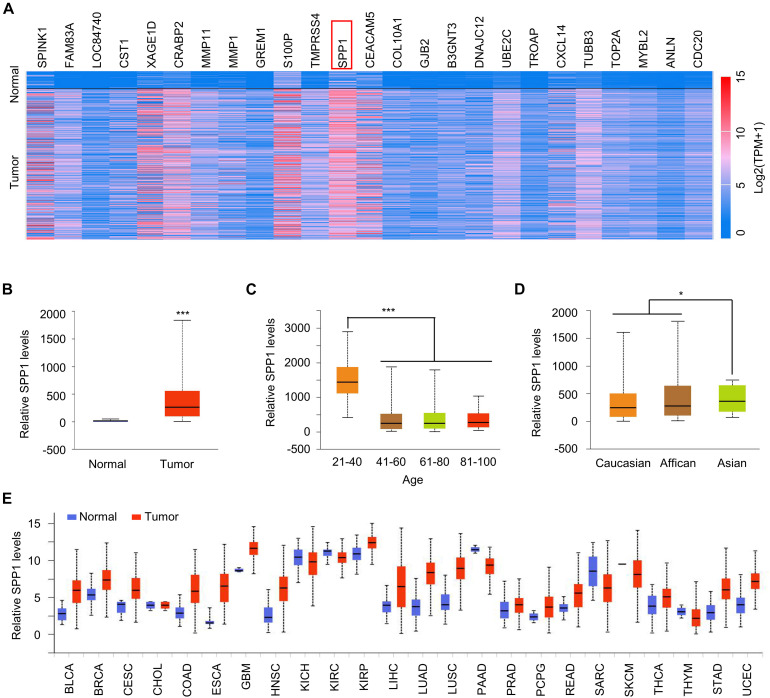
*SPP1* was upregulated in lung cancer. **(A)** Heatmap represented the top 25 over-expressed genes in lung cancer. **(B)** The *SPP1* levels in normal and lung tumor tissues was analyzed in TCGA datasets. **(C)** The *SPP1* levels in lung cancer patients with different age was analyzed in TCGA datasets. **(D)** The *SPP1* levels in different race of lung cancer patients was analyzed in TCGA datasets. **(E)** A pan-analysis of the *SPP1* expression in different cancer type. Data are shown as mean ± SD. *P < 0.05; ***P < 0.001; ns, not significant. Student’s *t*-test, or one-way ANOVA analysis.

We performed a genome-wide analysis of lung cancer tissues and corresponding normal tissues in the TCGA database and identified the top 25 genes that are highly expressed in lung cancer tissues. *SPP1* (isoform 5) upregulation showed the most pronounced upregulation in lung cancer tissues (Figure 1A). Further, *SPP1* expression was significantly higher in lung cancer tissues than in normal tissues (Figure 1B), and the expression of *SPP1* in young patients was also significantly higher than that in older patients (Figure 1C), which indicated that *SPP1* might be related to the rejuvenation of lung cancer. We also found that *SPP1* expression was higher in Asian patients than in other races (Figure 1D). To analyze whether the high expression of *SPP1* was specific in lung cancer or widely present in various tumors, we analyzed the data of multiple types of tumors, in addition to lung cancer, in TCGA and found that the expression of *SPP1* was significantly higher in most types of tumors than normal tissues. Figure 1E, which showed that *SPP1* might serve as a broad-spectrum tumor marker.

### High *SPP1* Correlates With Poor Survival Outcomes in Lung Cancer Patients

**FIGURE 2:**
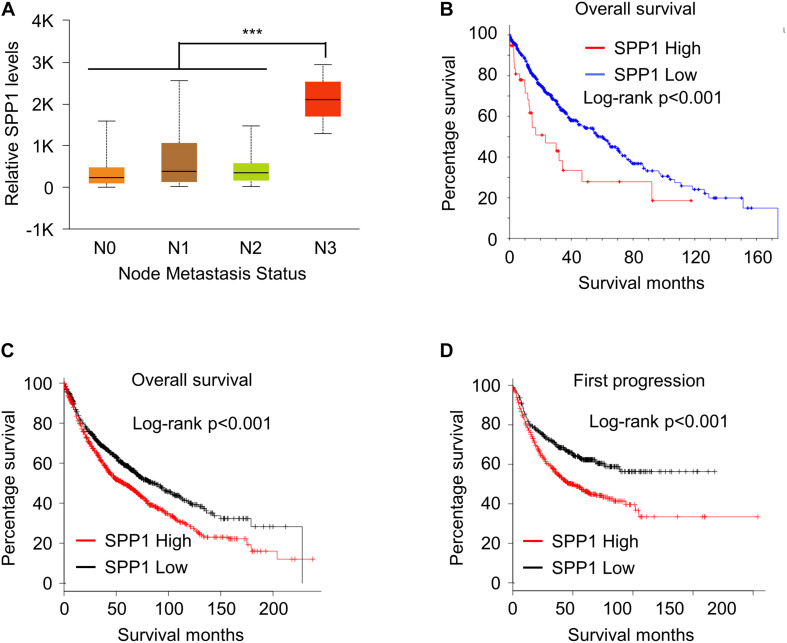
High *SPP1* correlated with poor survival outcomes in lung cancer. **(A)** The *SPP1* levels lung cancer patients with different node metastatic status was analyzed in TCGA datasets. **(B)** Kaplan–Meier plots of overall survival in lung cancer patients stratified according to their *SPP1* levels in TCGA datasets. **(C)** Kaplan–Meier plots of overall survival in lung cancer patients stratified according to their *SPP1* levels in GEO datasets. **(D)** Kaplan–Meier plots of first progression in lung cancer patients stratified according to their *SPP1* levels in GEO datasets. Data are shown as mean ± SD. ***P < 0.001; ns, not significant. One-way ANOVA analysis.

We next analyzed the correlation between the high expression of *SPP1* and the prognosis of patients with lung cancer. First, TCGA analysis revealed that the expression of *SPP1* was also higher in patients with high metastasis grades than in patients with no metastasis (Figure 2A). Further, patients with high expression of *SPP1* exhibited a poorer overall survival rate (Figure 2B). To further confirm this phenomenon, we analyzed the lung cancer patient data in the GEO database and found that patients with higher expression of *SPP1* had poorer overall survival rate (Figure 2C), and patients with higher expression of *SPP1* also had poorer first progression (Figure 2D). These data indicated that high expression of *SPP1* in lung cancer patients was correlated to poor survival of patients.

### *SPP1* Promotes Lung Cancer Cells Proliferation, Migration and Invasion

**FIGURE 3:**
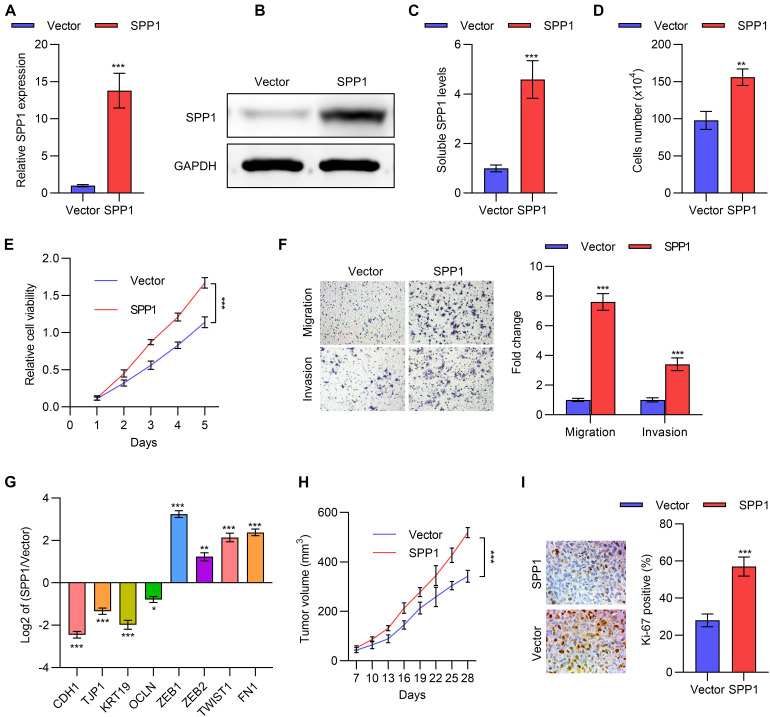
*SPP1* promoted lung cancer cells proliferation, migration and invasion. **(A)** The mRNA expression of *SPP1* in A549 cells transfected with *SPP1* expression plasmid was determined by qPCR. **(B)** The protein level of *SPP1* in A549 cells transfected with *SPP1* expression plasmid was determined by western blot. **(C)** The soluble *SPP1* levels in the supernatant of A549 cells transfected with *SPP1* expression plasmid was determined by ELISA. **(D)** Cell viability of A549 cells transfected with *SPP1* expression plasmid was determined by cell count assay. **(E)** Cell viability of A549 cells transfected with *SPP1* expression plasmid was determined by MTT assay. **(F)** Transwell of migration and invasion assay of A549 cells transfected with *SPP1* expression plasmid. **(G)** The mRNA expression of EMT markers in A549 cells transfected with *SPP1* expression plasmid was determined by qPCR. **(H,I)** The A549 cells transfected with *SPP1* expression plasmid was subcutaneously injected to nude mice. The tumor volume **(H)** and Ki67 **(I)** staining was determined. Data are shown as mean ± SD. *P < 0.05; **P < 0.01; ***P < 0.001; ns, not significant. One or two-way ANOVA test, Student’s *t*-test.

We next explored the function of *SPP1* in lung cancer at the cellular level. First, we constructed the *SPP1*-overexpressing cell lines by transfecting the *SPP1* overexpression plasmid into A549 and NCI-446 cells, and the overexpression was verified using qPCR and Western blot (Figures 3A,B and Supplementary Figure 1A). ELISA also confirmed *SPP1* overexpression in A549 cells after transfection (Figure 3C). Next, cell counting experiments and MTT assays were performed, which indicated that overexpression of *SPP1* in A549 and NCI-446 cells promoted cell proliferation (Figures 3D,E and Supplementary Figure 1B). Transwell experiments suggested that overexpression of *SPP1* could also promote the migration and invasion of A549 and NCI-446 cells (Figure 3F and Supplementary Figure 1C). This was accompanied by the down-regulation of epithelial markers and the up-regulation of mesenchymal markers as shown in Figure 3G. Further, we showed that *SPP1* overexpression in A549 cells promoted xenograft tumor growth in mice (Figure 3H). Further, as shown in Figure 3I, the Ki-67 staining of the tumor section suggested that the proliferative rate in the *SSP1*-overexpressing tumors was higher than the control group.

### *SPP1* Promotes Lung Cancer Cell Cisplatin Resistance

**FIGURE 4:**
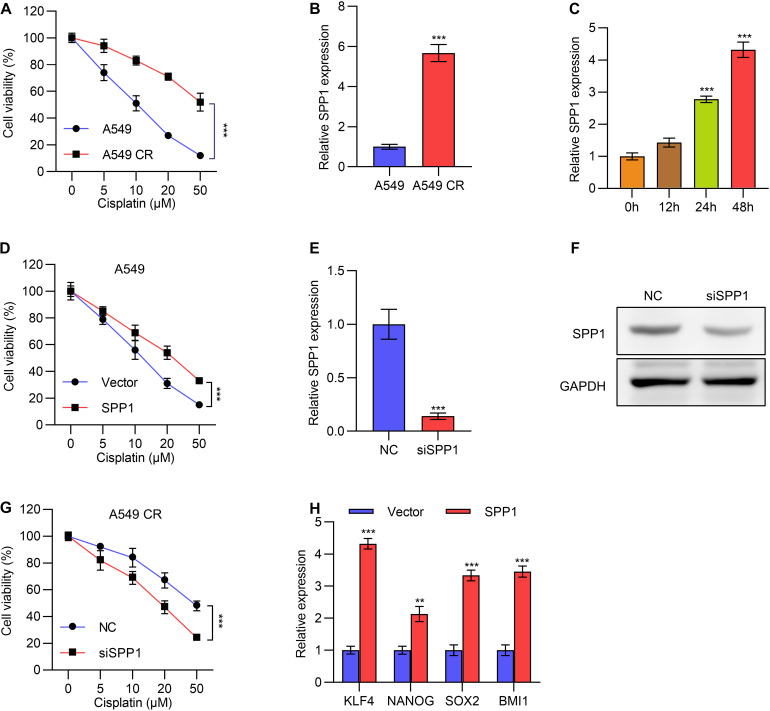
*SPP1* promoted lung cancer cells cisplatin resistance. **(A)** Cell viability of A549 or cisplatin resistant A549 CR cells treated with gradient concentration of cisplatin was determined by MTT assay. **(B)** The mRNA expression of *SPP1* in A549 or cisplatin resistant A549 CR cells was determined by qPCR. **(C)** The mRNA expression of *SPP1* in A549 cells treated with 10 μM cisplatin for different time was determined by qPCR. **(D)** Cell viability of A549 cells transfected with or without *SPP1* expressing plasmid and treated with gradient concentration of cisplatin was determined by MTT assay. **(E)** The mRNA expression of *SPP1* in A549 CR cells transfected with *SPP1* siRNA was determined by qPCR. **(F)** The protein levels of *SPP1* in NCI-446 CR cells transfected with *SPP1* siRNA was determined by western blot. **(G)** Cell viability of A549 CR cells transfected with or without *SPP1* siRNA and treated with gradient concentration of cisplatin was determined by MTT assay. **(H)** A series of stem cell markers in A549 cells transfected with *SPP1* expression plasmid was determined by qPCR. Data are shown as mean ± SD. **P < 0.01; ***P < 0.001; ns, not significant. One or two-way ANOVA test, Student’s *t*-test.

We next investigated the role of *SPP1* in the resistance to chemotherapy for lung cancer. We constructed cisplatin-resistant cell lines of A549 and NCI-446, named A549 CR and NCI-446 CR, respectively, and MTT experiments were performed to verify their resistance to cisplatin compared to parental control cells (Figure 4A). Next, we detected the expression of *SPP1* by qPCR, which showed that *SPP1* was significantly upregulated in drug-resistant A549 CR and NCI-446 CR cells (Figure 4B and Supplementary Figure 2A). A time-dependent upregulation of *SPP1* was also observed (Figure 4C and Supplementary Figure 2A) under treatment of 10 μM cisplatin.

We overexpressed *SPP1* in A549 cells and treated the cells with different concentrations of cisplatin. While higher concentrations of cisplatin led to lower viability, MTT assay showed that overexpression of *SPP1* could unsensitized A549 and NCI-446 cells to cisplatin (Figure 4D and Supplementary Figure 2B). Further, we knocked down the expression of *SPP1* with siRNA in drug-resistant A549 CR cells, and verified the knockdown efficiency by qPCR and Western blot experiments (Figures 4E,F). Expectedly, knocking down *SPP1* reduced drug resistance (Figure 4G). We next tested whether *SPP1* could promote the characteristics of tumor stem cells. Through qPCR detection, we found that overexpression of *SPP1* promoted the expression of a series of tumor stem cell markers (Figure 4H). Together, these data suggested that *SPP1* promoted cisplatin resistance in lung cancer, and targeting *SPP1* might be a potential treatment strategy to overcome resistance.

### The Expression of *SPP1* Is Regulated by DNA Methylation

**FIGURE 5:**
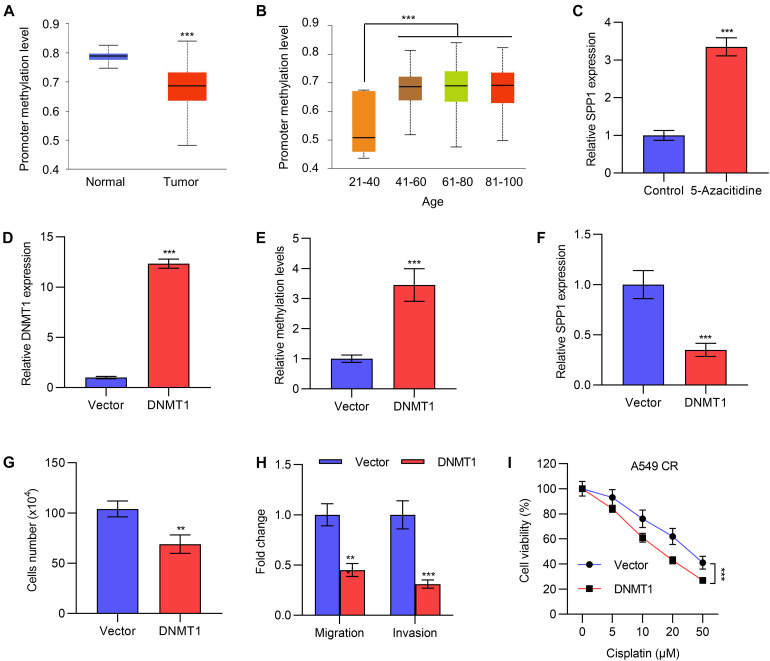
The expression of *SPP1* was regulated by DNA methylation. **(A)** The promoter methylation level of *SPP1* in normal and lung tumor tissues was analyzed in TCGA datasets. **(B)** The promoter methylation level of *SPP1* in lung cancer patients with different age was analyzed in TCGA datasets. **(C)** The mRNA expression of *SPP1* in A549 cells treated with 5-azacitidine was determined by qPCR. **(D)** The mRNA expression of *DNMT1* in A549 cells transfected with *DNMT1* expressing plasmid was determined by qPCR. (E) The methylation levels of *SPP1* in A549 cells transfected with *DNMT1* expressing plasmid was determined by sodium bisulfite assay. **(F)** The mRNA expression of *SPP1* in A549 cells transfected with *DNMT1* expressing plasmid was determined by qPCR. **(G)** Cell viability of A549 cells transfected with *DNMT1* expression plasmid was determined by cell count assay. **(H)** Transwell of migration and invasion assay of A549 cells transfected with *DNMT1* expression plasmid. **(I)** Cell viability of A549 cells transfected with or without *DNMT1* expressing plasmid and treated with gradient concentration of cisplatin was determined by MTT assay. Data are shown as mean ± SD. **P < 0.01; ***P < 0.001; ns, not significant. One or two-way ANOVA test, Student’s *t*-test.

To investigate the regulatory mechanism of *SPP1*, we performed TCGA analysis to compare the methylation levels of the promoter of *SPP1* in lung cancer tissues and normal tissues. We found that the methylation level of the *SPP1* promoter region in tumor tissues was significantly lower than that in normal tissues (Figure 5A), which might contribute to the high expression of *SPP1* in tumor tissues. Methylation levels were also significantly lower in young patients with high expression of *SPP1* than in older patients (Figure 5B). To validate this observation in cells, we treated the cells with a DNA methylation inhibitor 5-azacitidine, which indeed caused significant *SPP1* upregulation in lung cancer cells (Figure 5C and Supplementary Figure 3A).

DNA methylation is regulated by the DNMT family of methyltransferases, so we first overexpressed *DNMT1* by transfecting the expression plasmid of *DNMT1*, and verified its expression efficiency by qPCR experiments (Figure 5D). Sodium bisulfate assay also suggested that DNMT1 overexpression increased the methylation levels of *SPP1* in A5499 cells (Figure 5E). qPCR revealed that overexpression of *DNMT1* inhibited the expression of *SPP1* (Figure 5F and Supplementary Figure 3B), indicating a direct regulation of *SPP1* by *DNMT1*. Functionally, overexpression of *DNMT1* inhibited the proliferation, migration and invasion of A549 and NCI-446 cells (Figures 5G,H and Supplementary Figure 3C), as well as the resistance of A549 CR cells to cisplatin (Figure 5I), which was similar to the effects of *SPP1* knockdown. Taken together, the high expression of *SPP1* in lung cancer tissues was partly regulated by the reduced DNA methylation in its promoter region.

## Discussion

In the present study, we employed TCGA analysis and identified *SPP1* (isoform 5) as a cancer-promoting gene that contributes to poor prognosis, cancer progression and cisplatin-resistance of lung cancer. We specifically analyzed isoform 5 of *SPP1*, which is the longest transcript and therefore encodes the longest isoform. We showed that *SPP1* was the most pronouncedly upregulated gene in lung cancer, and patients with high *SPP1* expression demonstrated significantly poorer overall survival and first progression. Previously, *SPP1* has been reported to be upregulated in many cancer types (Dalla-Torre et al., 2006; Junnila et al., 2010; Huang et al., 2014; Qin et al., 2017). Indeed, our pan-analysis showed that a number of cancers were characteristic of *SPP1* upregulation. This evidence supports the use of *SPP1* as a broad-spectrum diagnostic and therapeutic in cancer.

The import role of *SPP1* in regulating the proliferation, migration, invasion and cancer resistance of lung cancer was validated in our study. Overexpression of *SPP1* was found to enhance cancer cell proliferation, migration and invasion, which were concomitant with downregulation of epithelial biomarkers and upregulation of mesenchymal biomarkers. This data suggests that *SPP1* is a driver of epithelial-to-mesenchymal transition (EMT), consistent with the cancer-promoting properties of *SPP1*. EMT is linked to the acquisition of invasiveness of cancer cells, thereby contributing to cancer metastasis and chemoresistance (Shih and Yang, 2011). In the meantime, *SPP1* overexpression promotes the expression of *KLF4*, *NANOG*, *SOX2*, and *BMI1*, which were cancer stem cell markers (Kaufhold et al., 2016). On the other hand, knockdown of *SPP1* using siRNAs effectively restored the sensitivity of A549 cells to cisplatin treatment, which suggests that silencing *SPP1* could be a viable means to increase the sensitivity of lung cancer to cisplatin treatment. This *SPP1* inhibition strategy has been employed in a recent study to overcome cisplatin resistance in cervical cancer (Chen et al., 2019). With the advance of siRNA delivery technologies (Oh and Park, 2009), the development of the *SPP1* silencing strategy could be a potential approach to improve the prognosis of lung cancer patients.

Epigenetic modification of genes is an important cause of abnormal gene expression in tumor cells, so we strived to know whether the abnormal expression of *SPP1* is also caused by changes in epigenetic modification (Yen et al., 2016). Here using TCGA analysis, we showed that lung cancer tissues were characteristic of hypomethylation in the promoter region of *SPP1*, which potentially contributed to the dysregulation of the *SPP1* gene. Further, the promoter methylation was found to be the lowest in young patients (age 21–40) compared to older patients, consistent with the finding that *SPP1* expression was the highest among young patients, which further supports hypomethylation of *SPP1* promoter as a mechanism of *SPP1* expression regulation. After adding 5-azacitidine, we show that *SPP1* expression was markedly upregulated, which was consistent with the function of 5-azacitidine in reducing DNA methylation (Tran et al., 2011). *DNMT1* encodes a maintenance methyltransferase to copy the DNA methylation patterns during DNA replication and repair. *DNMT1* is a member of the *DNMT*, showing *de novo*-type activity (Fuks et al., 2000). Upon overexpression of *DNMT1*, *SPP1* expression was prominently downregulated, accompanied with significantly attenuated cell viability, migration, invasion and cisplatin resistance. These data indicate that overexpression of *DNMT1*, which enhances DNA methylation, is another approach to suppress *SPP1* expression, therefore inhibiting lung cancer progression. Despite that *SPP1* is a well-known mediator of metastasis and a target for cancer therapeutics (Zhao et al., 2018), our data expand our knowledge on *SPP1* by suggesting that the high expression of *SPP1* in lung cancer is mainly due to its reduced methylation, and that *SPP1* can affect the metastasis of lung cancer cells as well as chemoresistance. The use of methyltransferase inhibitors can inhibit the expression of *SPP1* and thus suppress metastasis and drug resistance, providing a new approach for developing clinical treatment. However, it is worth noting that as DNMT1 methylates CpG sequence with no significant gene specificity, the overexpression of DNMT1 could induce a genome-wide DNA methylation similar as that by 5-AzaC. Therefore, we cannot exclude the possibility that DNA methylation indirectly regulates *SPP1*.

In conclusion, here we have demonstrated that *SPP1* is a cancer-promoting gene involved in lung cancer proliferation, migration, invasion and cisplatin resistance. *SPP1* upregulation is correlated with poor patient prognosis and survival. *SPP1* upregulation is promoted by hypomethylation of its promoter. Suppression of *SPP1* expression, either through siRNA or *DNMT1* overexpression, is a potentially novel approach to inhibit the progression of lung cancer.

## Data Availability Statement

The datasets presented in this study can be found in online repositories. The names of the repository/repositories and accession number(s) can be found in the article/Supplementary Material.

## Author Contributions

HT, JC, XH, YF, and FW conducted the experiments, analyzed the data, approved the publication of this manuscript, were accountable for all aspects of the work, and ensured the accuracy or integrity of any part of the work. HT and FW conceived the study and wrote the manuscript. All authors contributed to the article and approved the submitted version.

## Conflict of Interest

The authors declare that the research was conducted in the absence of any commercial or financial relationships that could be construed as a potential conflict of interest.
