# Supplementary material for: Upregulation of SPP1 Is a Marker for Poor Lung Cancer Prognosis and Contributes to Cancer Progression and Cisplatin Resistance
Source: Front Cell Dev Biol. 2021 Apr 29;9:646390. doi: 10.3389/fcell.2021.646390 (PMC8116663; doi:10.3389/fcell.2021.646390)
Supplement: Supplementary file 1 [file Data_Sheet_1.PDF]

Supplementary materials

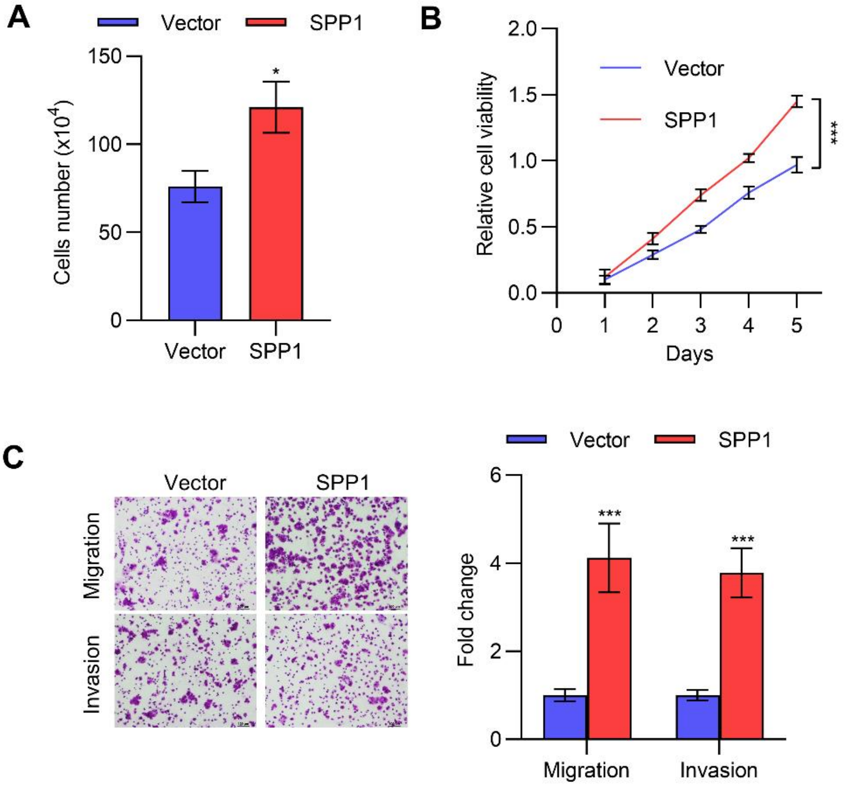

Figure S1. SPP1 promoted lung cancer cells proliferation and metastasis (Related to Fig. 3). (A) Cell viability of NCI-446 cells transfected with SPP1 expression plasmid was determined by cell count assay. (B) Cell viability of NCI-446 cells transfected with SPP1 expression plasmid was determined by MTT assay. (C) Transwell of migration and invasion assay of NCI-446 cells transfected with SPP1 expression plasmid. Data are shown as mean ± S.D. *P<0.05; **P<0.01; ***P<0.001; ns, not significant.

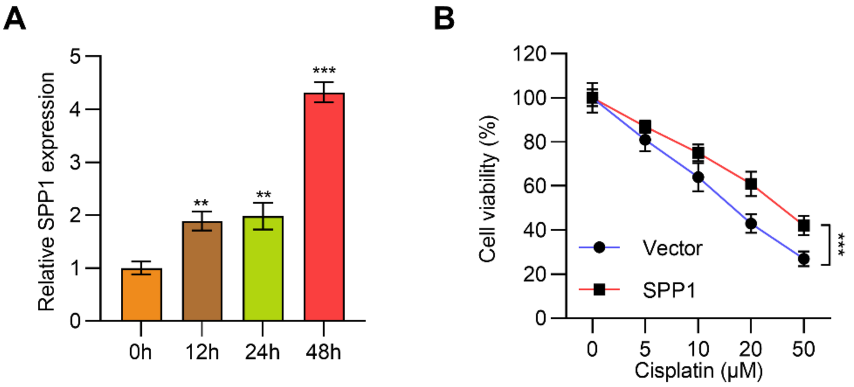

Figure S2. SPP1 promoted lung cancer cells cisplatin resistance (Related to Fig. 4). (A) The mRNA expression of SPP1 in NCI-446 cells treated with 10 μM cisplatin for different time was determined by qPCR. (B) Cell viability of NCI-446 cells transfected with or without SPP1 expressing plasmid and treated with gradient concentration of cisplatin was determined by MTT assay. Data are shown as mean ± S.D. *P<0.05; **P<0.01; ***P<0.001; ns, not significant.

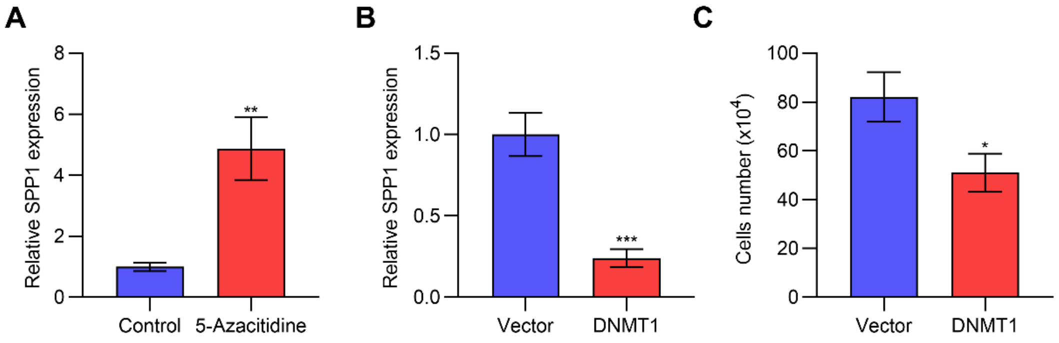

Figure S3. The expression of SPP1 was regulated by DNA methylation (Related to Fig. 5). (A) The mRNA expression of SPP1 in NCI-446 cells treated with 5-azacitidine was determined by qPCR. (B) The mRNA expression of SPP1 in NCI-446 cells transfected with DNMT1 expressing plasmid was determined by qPCR. (C) Cell viability of NCI-446 cells transfected with DNMT1 expression plasmid was determined by cell count assay. Data are shown as mean ± S.D. *P<0.05; **P<0.01; ***P<0.001; ns, not significant.
